# An optical coherence tomography and endothelial shear stress study of a novel bioresorbable bypass graft

**DOI:** 10.1038/s41598-023-29573-1

**Published:** 2023-02-20

**Authors:** Eric K. W. Poon, Masafumi Ono, Xinlei Wu, Jouke Dijkstra, Yu Sato, Matthew Kutyna, Ryo Torii, Johan H. C. Reiber, Christos V. Bourantas, Peter Barlis, Mohammed S. El-Kurdi, Martijn Cox, Renu Virmani, Yoshinobu Onuma, Patrick W. Serruys

**Affiliations:** 1 Department of Medicine, St Vincent’s & Northern Hospitals, Melbourne Medical School, University of Melbourne, Victoria, Australia; 2 Department of Cardiology, University of Galway, University Road, Galway, H91 TK33 Ireland; 3 Department of Clinical and Experimental Cardiology, Amsterdam UMC, Heart Center, Amsterdam Cardiovascular Sciences, University of Amsterdam, Meibergdreef 9, Amsterdam, The Netherlands; 4 Institute of Cardiovascular Development and Translational Medicine, The Second Affiliated Hospital, Wenzhou Medical University, Wenzhou, China; 5 Department of Radiology, Leiden University Medical Center, Leiden, The Netherlands; 6 CVPath Institute, Inc, Gaithersburg, MD USA; 7 Department of Mechanical Engineering, University College London, London, UK; 8 Institute of Cardiovascular Science, University College London, London, UK; 9 Department of Cardiology, Barts Heart Centre, London, UK; 10 Xeltis BV, De Lismortel 31, 5612AR Eindhoven, The Netherlands; 11 Emeritus Professor of Medicine, Erasmus University, Rotterdam, The Netherlands; 12 CÚRAM, SFI Research Centre for Medical Devices, Galway, H91 TK33 Ireland

**Keywords:** Cardiac device therapy, Biomedical engineering, Computational models

## Abstract

Endothelial shear stress (ESS) plays a key role in the clinical outcomes in native and stented segments; however, their implications in bypass grafts and especially in a synthetic biorestorative coronary artery bypass graft are yet unclear. This report aims to examine the interplay between ESS and the morphological alterations of a biorestorative coronary bypass graft in an animal model. Computational fluid dynamics (CFD) simulation derived from the fusion of angiography and optical coherence tomography (OCT) imaging was used to reconstruct data on the luminal anatomy of a bioresorbable coronary bypass graft with an endoluminal “flap” identified during OCT acquisition. The “flap” compromised the smooth lumen surface and considerably disturbed the local flow, leading to abnormally low ESS and high oscillatory shear stress (OSI) in the vicinity of the “flap”. In the presence of the catheter, the flow is more stable (median OSI 0.02384 versus 0.02635, p < 0.0001; maximum OSI 0.4612 versus 0.4837). Conversely, OSI increased as the catheter was withdrawn which can potentially cause back-and-forth motions of the “flap”, triggering tissue fatigue failure. CFD analysis in this report provided sophisticated physiological information that complements the anatomic assessment from imaging enabling a complete understanding of biorestorative graft pathophysiology.

## Introduction

The burden and extent of coronary artery disease (CAD) and the clinical presentation determine the optimal treatment strategy with either percutaneous or surgical revascularisation. Coronary artery bypass grafting (CABG) is preferred in complex multi-vessel lesions and diabetic patients, given its longer beneficial effect where a biocompatible conduit is used to bypass the diseased vessel and restore myocardial blood flow. While CABG is an effective treatment for CAD, graft failure resulting from thrombosis, atherosclerosis and intimal hyperplasia (IH) remains a concern^1^. Several studies have shown that arterial grafts have higher patency than venous grafts^2,3^. For example, radial artery grafts show an excellent patency rate of 98.3% 5 years after surgery^4^. Conversely, approximately 10–15% of saphenous vein grafts (SVGs) fail in their first year with their failure rate rising to 50% at 5 years post-surgery^5,6^. Despite SVGs’ lower patency rate, they are still routinely deployed due to the limited availability of arterial grafts. Lastly, the 1-year patency rate for a polymer (polytetrafluoroethylene) conduit is around 60%. It declines sharply to only 32% 2 years post-surgery^7^.

Tissue-engineered vascular grafts, made of allogeneic smooth muscle cells and seeded with autologous endothelial cells, represent the latest development in artificial bypass grafts which have demonstrated a 100% patency rate for 30 days in canine coronaries^8^. Extensive research efforts have targeted the optimal biological and/or biomechanical conduit for use with CABG^9–11^. Recently, we have investigated the medium-term patency of a biorestorative coronary artery bypass graft (XABG, Xeltis BV, Eindhoven, the Netherlands) and examined the implications of four key biomechanical factors [bending angles, superficial wall strain, endothelial shear stress (ESS) and delta (segmental) quantitative flow ratio ΔQFR)] derived from three-dimensional quantitative coronary angiography (3D-QCA) on graft restenosis over a period of 1, 3, 6, 9, and 12 months^12,13^.

This is a case study examining the flow patterns in a 3D XABG model in a challenging sheep model. The bypass graft was patent until 6 months follow-up. Late graft alterations were observed at 6 months with high-resolution intravascular optical coherence tomography (OCT) imaging. Graft alteration in the form of a 3D tissue “flap” within the conduit was presumably due to the interaction between the graft and catheter. Such 3D structure within the conduit can be challenging to model with 3D-QCA alone. OCT provides exceptional details of the non-uniform endoluminal lining. When OCT is fused with 3D-QCA, the three-dimensional graft reconstruction (termed 3D-QCA-OCT hereinafter) model represents a high-precision and reliable model graft geometry for detailed assessment of the impacts on local haemodynamic performance. To the best of our knowledge, this study represents the first report describing *in-vivo* the geometrical complexity of a 3D endoluminal “flap” and the haemodynamic forces on this novel biorestorative supramolecular polymer bypass graft.

## Results

**Figure 1:**
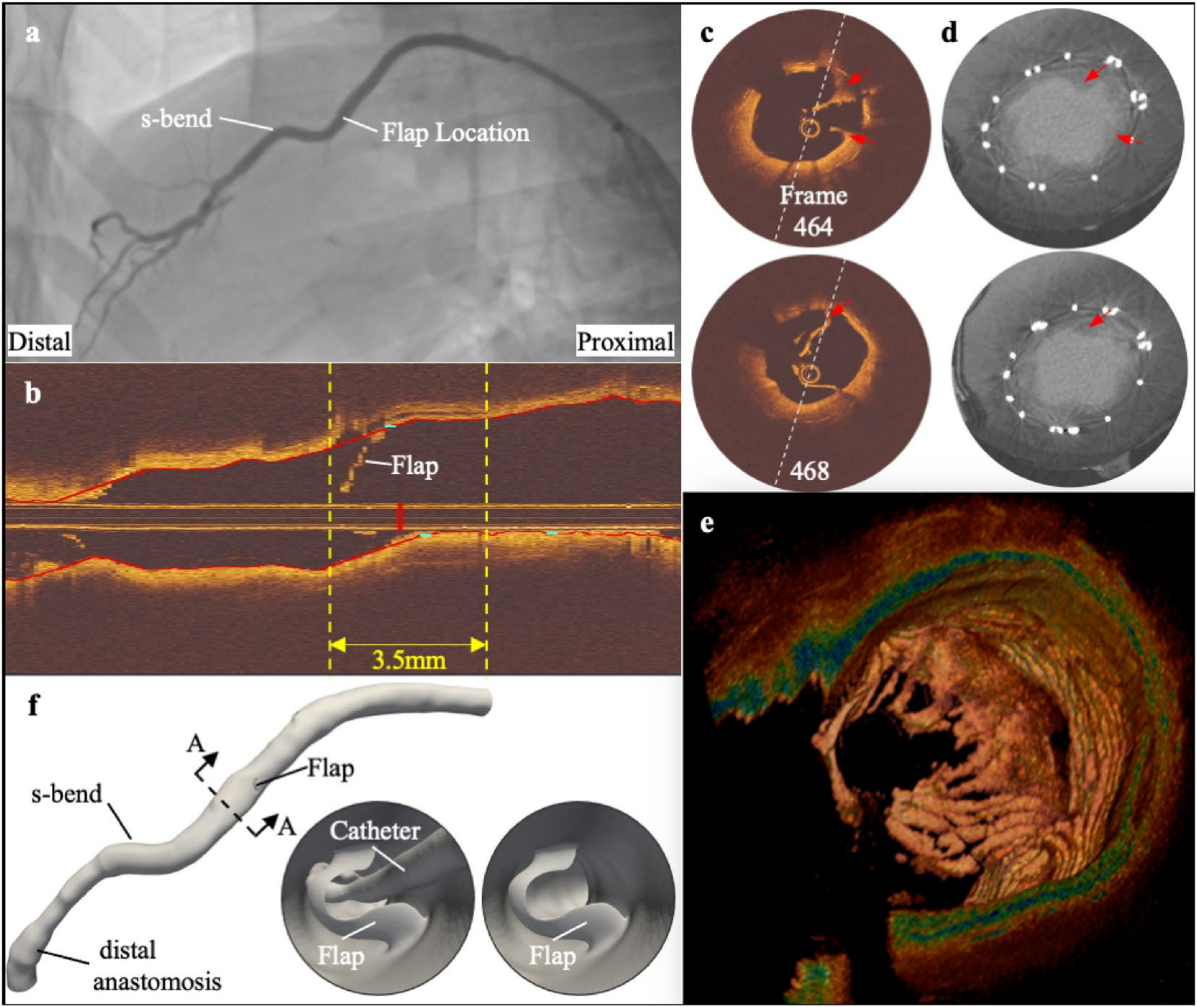
Routine imaging acquisitions (coronary angiogram and OCT) at 180 days and µCT at 200 days. (**a**) 2D angiographic views of biorestorative polymeric coronary bypass grafts and the two consecutive OCT pullback locations. (**b**) The two OCT pullbacks were manually matched and combined to form a single longitudinal view. The “flap” covers a length of 3.5 mm (35 frames), as indicated by the yellow dashed lines. (**c**) Selected 2D OCT cross-sections within the “flap” segment. The white dash line indicates the circumferential location of the longitudinal view in (**b**). (**d**) displays co-registered µCT images at 200 days. Red arrows point to the remaining shoulders of the “flap”, which was originally identified at 180 days OCT images. (**e**) 3D OCT representation of the “flap”. (**f**) 3D computer-aided design model of the bypass graft using the 3D-QCA-OCT reconstruction technique. A section view A–A (with distal segment removed at black dashed line) shows the internal view of the “flap” with (and without) the OCT catheter. *µCT* micro-computed tomography, *OCT* optical coherence tomography, *QCA* quantitative coronary angiography.

A total of 1500 OCT frames from three pullbacks were analysed at 100 µm intervals from sequences obtained 6 months following the XABG implantation. The proximal pullback was excluded from the 3D artery reconstruction due to the inability to co-register these data (mismatch in lumen area) with 3D-QCA. The distal and middle OCT pullbacks were combined into a single pullback by discarding 145 overlapping frames. The combined OCT frames (n = 931) were used for 3D reconstruction. Graft delamination or possibly iatrogenic subintimal dissection was noted in longitudinal view (Fig. 1b) and in 35 cross-sections (i.e., 3.5 mm in length) (Fig. 1c). The detached tissue created a flow divider like geometrical feature (see Fig. 1c,e; Supplementary Video S1). 3D reconstruction of this feature revealed a “flap” like appearance structure that was nearly perpendicular to the lumen (Fig. 1f). The conduit also displayed an “s-shape” bend immediately distal to the “flap” (Fig. 1a,f). At 200 days follow-up, micro-computed tomography (µCT) images showed similar tissue remained at the shoulder of the “flap” (Fig. 1d; red arrows). However, the tip of the “flap” was no longer present. Nevertheless, the conduit remained patent at 200 days despite the presence of the “flap” (see Fig. S1).

**Figure 2:**
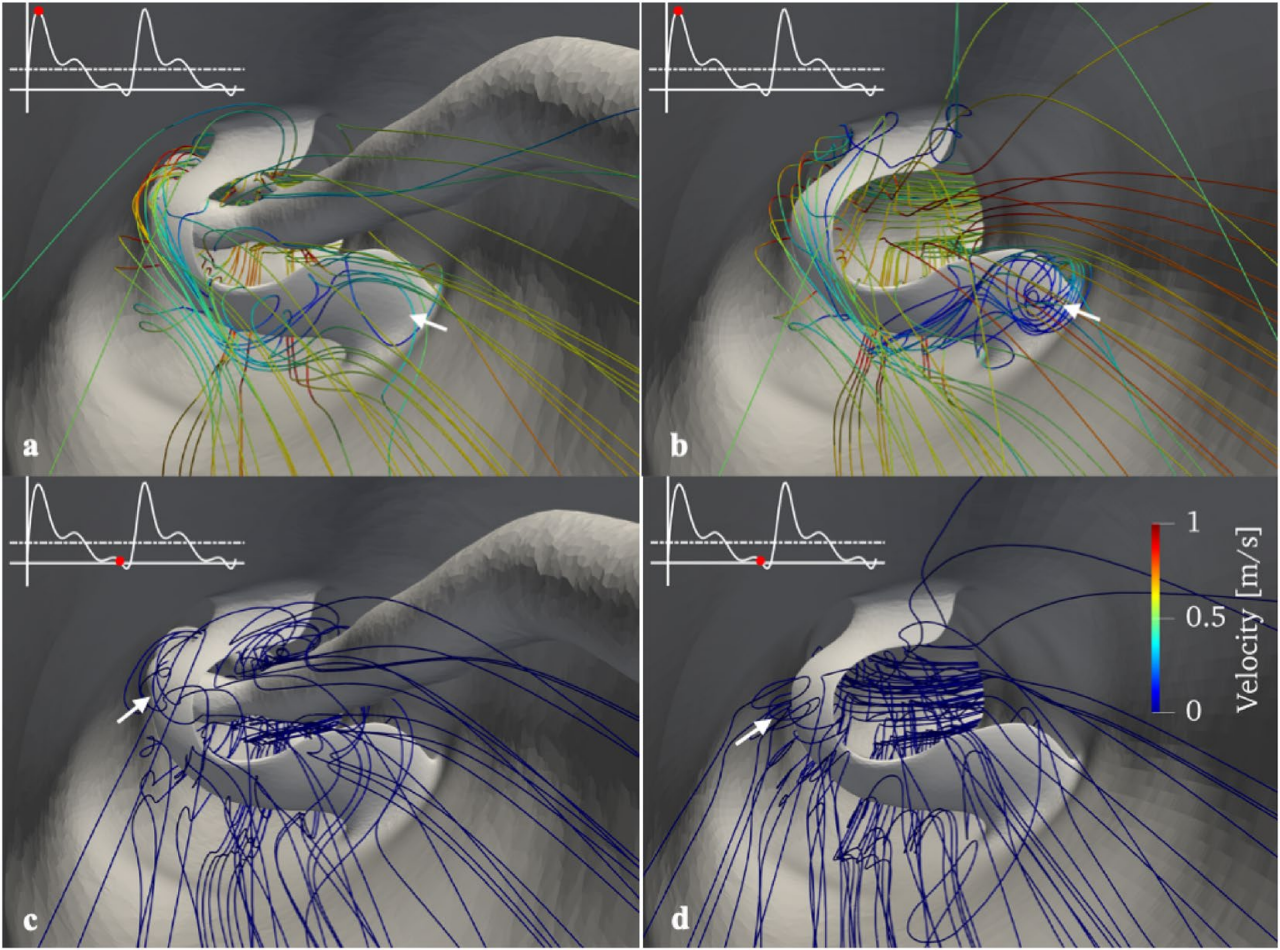
Section view A–A (Fig. 1f) with flow pattern distal to the “flap” with (left panels) and without the catheter (right panels). Results are shown at peak blood flow velocity (**a,b**) and minimum blood flow velocity (**c**,**d**). White arrows point to the flow recirculation distal to the flap.

The “flap” was a major obstruction to the flow (Fig. 2). Regardless of the phase within a cardiac cycle, flow reversal was observed distal to the “flap”. The haemodynamic environment changed substantially between peak and minimum flow. At peak flow, the flow reversal was most pronounced at the interface between the “flap” and lumen surface of the graft (white arrow in Fig. 2a,b) and disappeared when the flow was minimum. On the other hand, flow reversal dominated on the tip of the “flap” at minimum flow (Fig. 2c,d; white arrow) and was dissipated during peak flow. Removing the catheter from the XABG model allowed more flow through the “flap” opening, resulting in a more pronounced flow reversal pattern.

**Figure 3:**
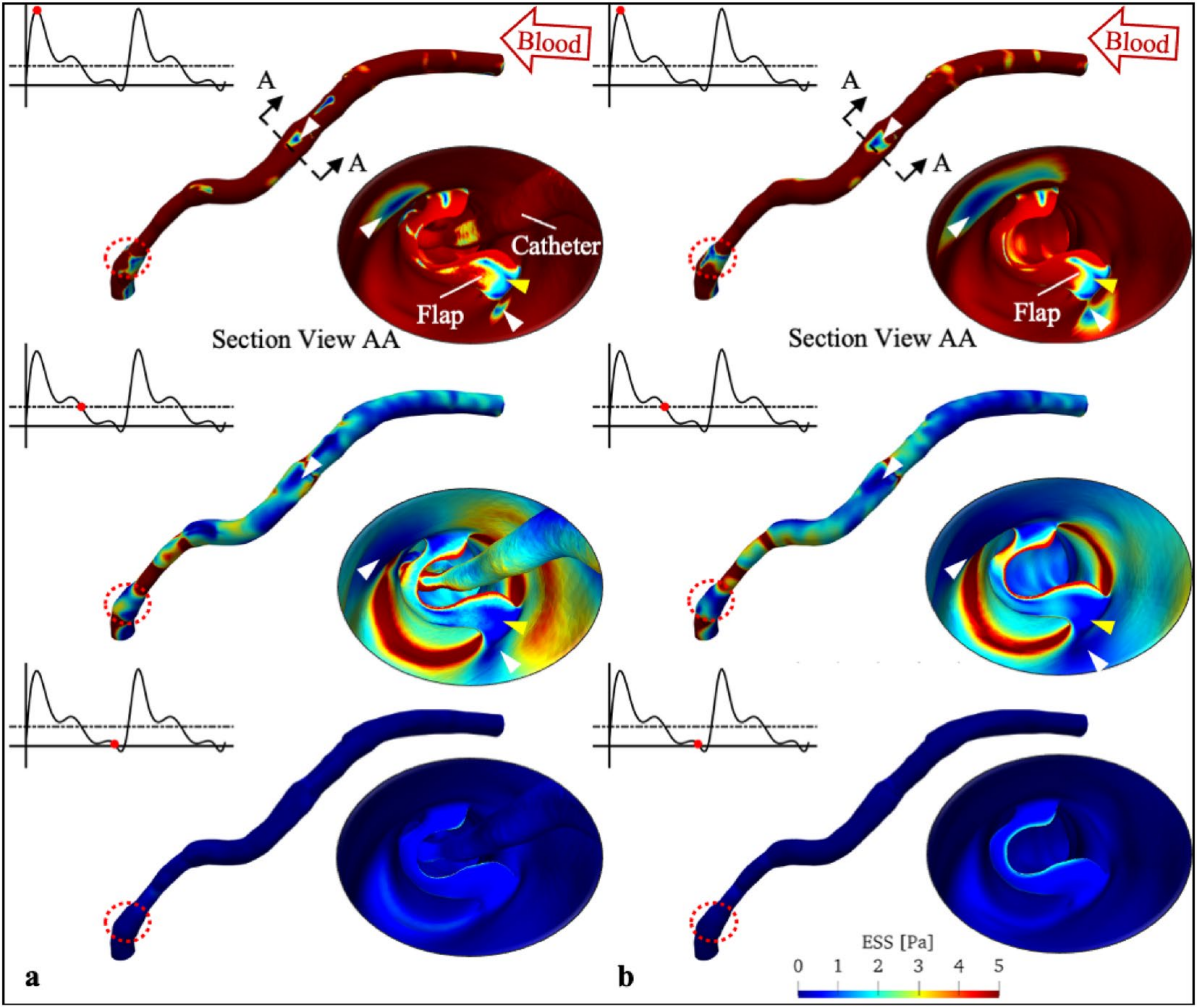
ESS patterns on the XABG at selected angiogram frame time points: peak (time pt. 2), median [time pt. 6 (dashed line)] and minimum blood flow (time pt. 9). Insets show the section view A–A and the corresponding ESS pattern distal to the “flap”. White and yellow arrows point to the low ESS region on the graft lumen and “flap” surface, respectively. Red dotted circles highlight the focal region of low ESS at distal anastomosis. (**a**) CFD with the OCT catheter and (**b**) CFD without the OCT catheter. *CFD* computational fluid dynamic simulation, *ESS* endothelial shear stress, *OCT* optical coherence tomography, *XABG* Xeltis coronary artery bypass graft.

Figure 3 shows the ESS patterns on the XABG at elected time points. These time points correspond to the peak blood flow velocity, the median and the minimum blood flow velocity measured from the contrast-filled graft centreline. Regardless of the CFD simulations with or without the OCT catheter, focal regions of low ESS were noted on the distal surface of the “flap” and graft luminal surface (see yellow and white arrows in insets of Fig. 3). In contrast, high ESS was shown on the proximal “flap” surface (see Supplementary Fig. S1). The influence of the catheter on the ESS distribution depends strongly on the region of interest (ROI) and the phase of the cardiac cycle. (Supplementary Table S2 and S3).

**Figure 4:**
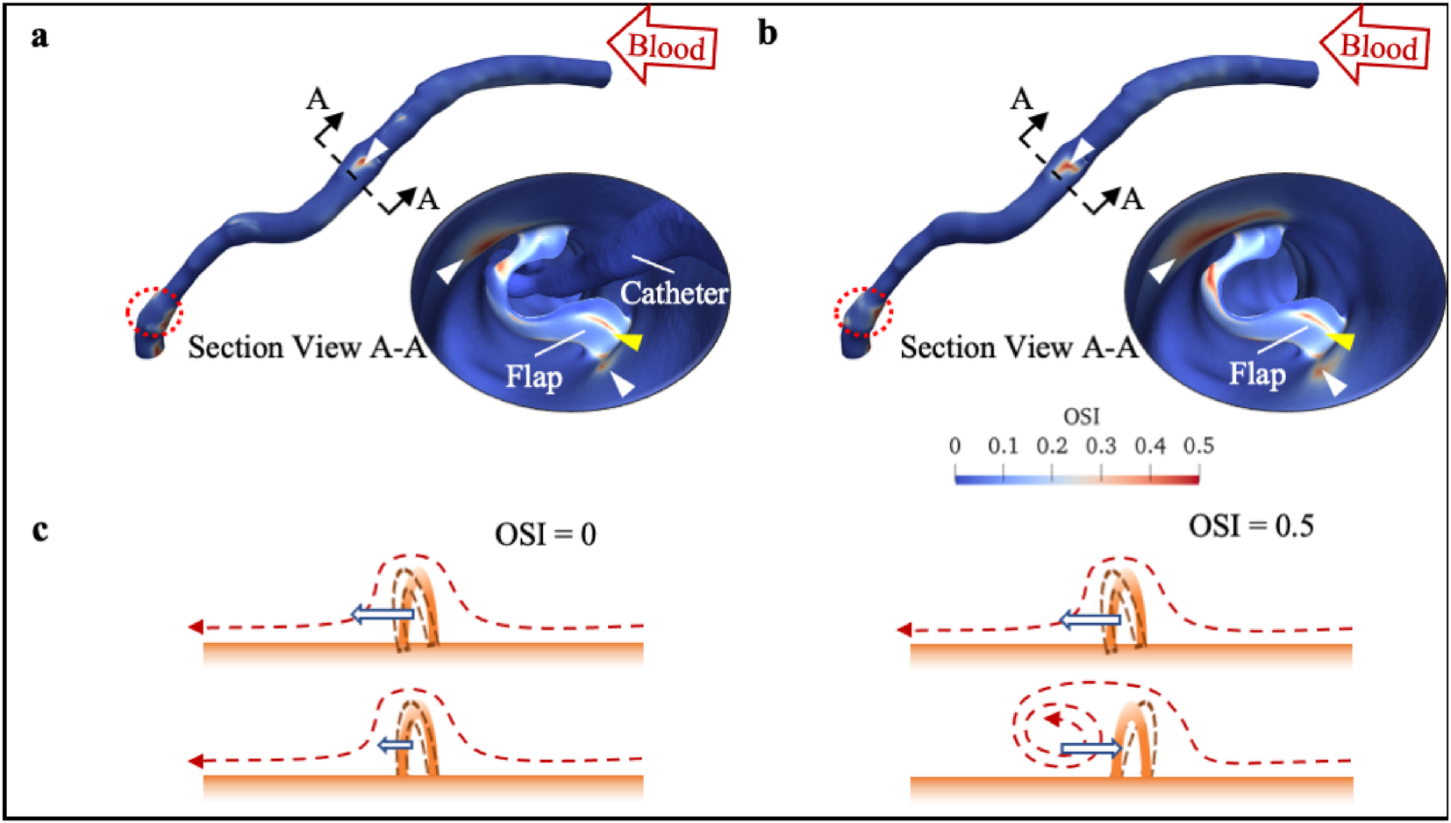
OSI patterns on the XABG: (**a**) with and (**b**) without the catheter. Insets show section views A–A and the corresponding OSI patterns distal to the “flap”. Red dotted circles highlight the focal region of high OSI at distal anastomosis. (**c**) Cartoon showing the impact of different OSI on the flap. *OSI* oscillatory shear index. *XABG* Xeltis coronary artery bypass graft.

Figure 4 compares the OSI with and without the catheter. High OSI was observed distal to the “flap” both with and without the OCT catheter (Fig. 4a,b; white arrows in insets). Once the catheter was removed, OSI of the bypass graft increased significantly (Supplementary Table S3). Median OSI increased by more than 1.5 times within the distal 3 mm ROI without the OCT catheter (Supplementary Table S4), while in both the “flap” and proximal 3 mm ROIs there was a reduction in median OSI in the absence of the OCT catheter.

Figures 3 and 4 (red dotted circles) also reveal focal regions of low ESS and high OSI at the distal anastomosis. Both areas of low ESS and high OSI increased as the catheter was withdrawn. Moreover, the maximum OSI on the XABG was found at the distal anastomosis (0.4842 with the catheter and 0.4860 without the catheter) instead of near the flap (0.4612 and 0.4837 respectively) or at the “flap” surface (0.4818 and 0.4674 respectively).

## Discussion

This study describes the immediate effect in the haemodynamic environment during vessel interrogation with an OCT catheter that has transfixed an endoluminal “flap” identified by OCT at 6 months follow-up in a synthetic bioresorbable graft implanted in an ovine model. The in-silico results were further correlated with µCT findings 3 to 4 weeks later (at 200 days). The study presents an in-vivo assessment of the local haemodynamic micro-milieu in a challenging vessel geometry (e.g., endoluminal dissection, graft delamination, etc.) that affected flow patterns considerably. Our results demonstrated a substantial increase in flow disturbance in the endoluminal “flap” vicinity that might be associated with luminal narrowing and potential graft degradation. This highlights that (1) OCT imaging (given its superior resolution) is essential in evaluating the short and long-term performance of emerging novel devices and therapies, (2) OCT enables comprehensive and detailed CFD analysis leading to a more accurate assessment of the implications of the local haemodynamic force distribution of device performance, and (3) although it would equally suggest that OCT imaging carries additional procedural risk, this advanced imaging modality can help assess bioengineered grafts.

At the time of OCT imaging at 6 months, an endoluminal “flap” was discovered. The discovery of the “flap” raised a series of clinical concerns. Firstly, it is unclear whether the “flap” found by OCT was spontaneous or iatrogenic and whether it represents subintimal delamination or dissection. Unfortunately, the answer to this question will never be known since OCT imaging was performed at a single time point. We cannot discount that the “flap” might have been induced during the intravascular OCT procedure, thereby compromising the integrity of the XABG. Yet, a recent study comparing 1142 OCT procedures versus 2476 intravascular ultrasound procedures demonstrated that incident rates were rare for both modalities (0.6% and 0.5%, respectively, p = 0.60)^14^. Although it is important to reveal the sequent of events that led to the presence of the endoluminal “flap” from a clinical and device manufacturer’s standpoint, it is equally (if not more) important to monitor the impacts of the “flap” on the surrounding haemodynamic environment.

Haemodynamics plays a vital role in the conduit’s patency. The presence of the catheter worsened the situation by holding the “flap” in place as evidenced by the catheter-size hole in Fig. 1e. However, it is unclear whether the “flap” would retain its geometry once the catheter was withdrawn. In the best-case scenario, the “flap” might reattach to the lumen surface with minimum haemodynamic disturbance. In contrast, if the “flap” maintained its geometry, this worst-case scenario would pose the greatest disturbance to the nearby haemodynamic. Furthermore, the relatively thin “flap” at the tip would allow for some degrees of movement which might affect shear stress assessment and hence result in additional technical challenges.

Our final concern was whether the graft was at risk of thrombosis due to flow disturbances caused by this “flap”. Should the animal require additional intervention with anticoagulant drugs? These questions are important considerations given the limited number of animals studied in this study. We tried to answer some of these aforementioned concerns with a sophisticated ESS assessment. We evaluated their impact on the changes in lumen complex geometry and co-registered our findings with µCT at 200 days follow-up.

We observed that portions of the “flap” remained at 200 days shown by µCT (Fig. 1d; red arrows). This corresponds to the “flap” shoulder regions that reflect the thickest component of the “flap” as displayed by the 6 months OCT (Fig. 1d; red arrows). The fate of the other remanent of the “flap” remains unclear and might reflect a process of reabsorption or distal embolisation over time.

A smooth laminar flow in a conduit is regarded as the ideal haemodynamic environment as this is associated with homogeneous physiological ESS^15^. Under these favourable conditions, the endothelial cells are in an elongated fusiform arrangement and have athero-protective function^16^. Any obstruction (e.g., catheter, “flap”, etc.) in the conduit could seriously jeopardise the desired laminar blood flow, leading to rapid fluctuations in flow velocity and flow oscillations.

The effects of these local blood disturbances at the “flap” and its vicinity are twofold. On the lumen, local flow oscillations led to adversely low ESS at both shoulders of the “flap” (Figs. 3 and 4; white arrows). This results in reduced p-eNOS and KLF-2 expression, increased VCAM-1 expression, Ox-LDL uptake, cytokine-induced monocyte adhesion, and tissue factor, increasing the risk of vulnerable plaque formation and thrombosis^17^.

In contrast, a different effect/mechanism was postulated on the “flap’s” surface. The “flap” was positioned almost perpendicularly to the main flow direction, and this resulted in flow recirculation distal to the “flap” (Fig. 2; white arrows). Moreover, flow reversal was also noted at both maximum and minimum flow rates once the catheter was withdrawn (Fig. 2b). The flow reversal was also reflected by an increase in maximum OSI [0.4612 (with catheter) versus 0.4837 (without catheter)] on the “flap” surface (Fig. 4a,b). OSI indicates the oscillation of the local flow; it quantifies the back-and-forth local flow motion^18^. Zero OSI signifies a constant unidirectional flow motion, whereas OSI approaching 0.5 indicates strong locally back-and-forth oscillating flow (Fig. 4c). More importantly, when co-registering the µCT images at 200 days (Fig. 1d) with our CFD results (Fig. 2a,b), we have demonstrated an increase in oscillatory flow activities in the vicinity of the “flap”. Oscillating flow on the “flap” poses an increased risk of material fatigue by bending the “flap” back-and-forth repeatedly. In addition, the increased oscillatory flow around the “flap” resulted in a slower flow movement. Contrary to popular belief, the slow flow might accelerate the bioresorption rate of the “flap”^19^.

Shear stress assessment might also provide insights into surgical anastomosis techniques. As demonstrated in Figs. 3 and 4, CFD also identified abnormal low ESS and high OSI at the distal anastomosis. Our CFD results showed that the highest OSI was not at the “flap” but at the distal anastomosis (0.4674 versus 0.4860). Similar to the oscillatory flow environment observed near the “flap”, it is hypothesised that these low ESS and high OSI conditions could lead to vulnerable plaque and thrombosis formation^17^. Thus, CFD might play a role in future surgical planning with pre-surgical virtual haemodynamic analysis and optimisation.

### Limitations

This study has several limitations. First, it is assumed that the “flap” stayed in place after the withdrawal of the OCT catheter. Although the shape and position of the “flap” were partially supported by the 200 days µCT images, the relatively thin “flap” at the tip would still allow for some degrees of movement, hence affecting shear stress assessment. However, a comprehensive investigation of the “flap” motions will require a multidisciplinary approach involving fluid–structure interaction (FSI) methods. Although FSI has been widely employed to analyse the shear stress around aortic valves, it requires solving additional equations for the motion of the object of interest^20,21^. More importantly, this equation has to simultaneously handle the solid mechanic properties of the object of interest as well as the coupling effects between its motion and the transient haemodynamic environment. Hence, FSI represents a significant numerical challenge^22^.

Furthermore, while there are ample *in-vivo* and *in-vitro* data for validation FSI modelling of the aortic valves, comprehensive descriptions of detailed flow structures around the aortic valves are still rare^22^. Thus, adding FSI to the present study would introduce additional uncertainties to our analysis.

Secondly, although our CFD findings may support a distal embolisation in the case of material fatigue or a faster resorption of the flap due to the presence of flow statis next to the “flap”, the exact mechanism responsible for the missing “flap” in µCT images at 200 days is unknown. Besides, with the “flap” missing within a short period (20 days) between OCT imaging and µCT, we should not discount the possibility of both mechanisms acting simultaneously on the “flap”. In other words, an accelerated resorption process that led to an earlier fatigue failure. A material cross-sectional analysis of “flap” shoulder remains (Fig. 1d) might provide additional insight into the “flap” failure mode^23^, and should be recommended for further investigation in a similar event.

In terms of CFD analysis, one of the limitations was that the flow rate and other physiological conditions with and without the catheter were assumed to be constant. In addition, CFD findings at the distal anastomosis should be interpreted with caution due to the lack of long-term follow-up data. While early CFD results might have identified an interesting haemodynamic observation at the distal anastomosis, large-scale prospective trials are warranted to evaluate these early findings.

## Conclusion

Modern graft technologies have been introduced to optimise clinical outcomes in patients undergoing bypass operations. In this hypothesis-generating study, we demonstrated the importance of a detailed assessment of graft morphology using serial imaging (OCT and µCT) and CFD analysis which allows an in-depth evaluation of the morphological changes and physiological characteristics of these technologies. This information is expected to help optimise conduit design before its application in clinical practice.

## Methods

### Study design

A biorestorative XABG made from a supramolecular polymer was implanted in an ovine model. The XABG was electrospun from polyester urethanes that contained the ureidopyrimidinone supramolecular binding motif^24^, and had an embedded nitinol microskeleton for kink resistance. The bypass graft has a luminal diameter of 4 mm and is approximately 150 mm in length. The study was conducted following the ARRIVE guidelines (Animal Research: Reporting of In Vivo Experiments), the Guide for Care and Use of Laboratory Animals approved by the local Institutional Animal Care and Use Committee. The study also was approved by the Test Facility’s Ethical Committee for compliance with regulations prior to study initiation (Protocols IQI001-IS02 and IQI005-IS02).

The ovine model was anesthetised with an appropriate barbiturate by IV administration. A left lateral thoracotomy was performed to allow access to the heart and descending aorta. Cannulas were placed for cardiopulmonary bypass (CPB). The left anterior descending artery and the descending aorta were isolated and assessed for appropriate distal and proximal anastomoses. The distal anastomosis was checked for haemostasis and clamped to prevent haemorrhage while the aorta was punctured and anastomosed to the proximal end of the graft. After appropriate haemostasis of the proximal anastomosis, the graft was de-gassed, and clamps were removed. Once complete haemostasis was achieved and the animal was stable, CPB cannulas were removed and all incisions were routinely closed. Protamine sulfate was administered as needed to control any haemorrhage associated with the procedure.

### Angiography and optical coherence tomography

Coronary angiography and OCT imaging were performed at 6 months through the femoral artery. The vessel was punctured under general anaesthesia, and a 5-French sheath was inserted. Selective bypass angiography from the ostium of the graft in the descending aorta was performed with a JR 4.0 catheter. Nitrates were given before the angiographic injections. Cine images were recorded at 15 frames/s. Two angiographic runs in the anterior–posterior and lateral views were obtained and converted to DICOM (Digital Imaging and COmmunications in Medicine) format at a resolution of 512 × 512 pixels. Angiograms showed a mild stenosis in the middle XABG segment (Fig. 1a). Therefore, the operator proceeded to OCT imaging to assess this lesion.

Optical coherence tomography (OCT) was performed using the Dragonfly Optis imaging catheters and Optis Mobile System (Abbott Vascular, Westford, MA). Under fluoroscopic guidance, the OCT catheter was advanced into the graft, and the radio-opaque distal marker of the OCT catheter was positioned distally to the anastomosis between the bypass graft and the native coronary artery. Automated pullback was performed under contrast agent injection. Since the pullback is too short to image the entire graft at once, the graft was split into an overlapping distal, mid and proximal part.

### Micro-computed tomography (µCT) analysis

Given the uniqueness of the OCT finding on the endoluminal “flap” at 6 months, the animal was electively sacrificed approximately 3 to 4 weeks after the 6 months OCT follow-up (at 200 days). This allowed us to gain access to the bypass graft for detailed analyses. The mid-graft segment was fixated in formalin, dissected and scanned with µCT at 34 µm voxel. Each µCT image had a 25.6 mm width and 27.7 mm height. Over 4,000 sections were generated and co-registered and compared with the OCT images at 6 months.

### Three-dimensional XABG reconstruction

OCT pullback revealed a tissue obstruction in the distal to middle segment (Fig. 1b,c) that formed a “flap” perpendicular to the long axis of the XABG (Supplementary Video S1). For this reason, 3D reconstruction of the XABG was performed by fusing the 3D-QCA centreline with accurate anatomy from OCT images. The reconstruction was carried out in the following steps. The 3D centreline of the graft was first reconstructed from two end-diastolic projections using the validated QAngioXA 3D RE software (Medis Medical Imaging bv, The Netherlands)^25^. Post-hoc synchronisation between two projections was performed by ECG superimposed on the fluoroscopic images. In the second step, XABG contours (including the conduit lumen border, the “flap” geometries and the OCT catheter border) were semi-automatically delineated and manually corrected as necessary on each OCT frame by two expert analysts using QCU-CMS software (Leiden University Medical Center, The Netherlands). Matching of OCT frames was performed in a two-step process: (i) visually identify the tip, lens, and proximal markers of consecutive pullbacks and (ii) fine-tune overlapping segments by matching lumen cross-sectional area including rotation of the entire pullback if needed. Multiple OCT pullbacks were combined, to form a single 3D reconstruction.

Each set of the delineated OCT contours (lumen, ‘flap’, and the catheter) was placed perpendicularly onto the 3D centreline using a validated in-house algorithm (MATLAB R2020b, Mathworks Inc., Natick, MA)^26^. The axial orientation of each frame was estimated using distal anatomical landmarks or the lumen silhouette (and area) from 3D-QCA^27,28^. Rotational matching was first performed using distal anatomical landmarks. The frame-to-frame relative twist within the bypass along the 3D centreline was accounted for using the sequential triangulation algorithm^29^. Three-dimensional computer-aided design models of the bypass graft, the “flap”, and the OCT catheter were individually reconstructed and exported in STL (stereolithography) format for further processing (such as superimposing the flap through a series of Boolean operators, including the OCT catheter, etc.) using Autodesk Meshmixer (v3.5 Autodesk Inc. San Rafael, US) (Fig. 1e).

### Computational fluid dynamics analysis

Computational fluid dynamics investigations on the 3D-QCA-OCT models were carried out according to the expert recommendations^30^. Briefly, the lumen model derived by the fusion of 3D-QCA and OCT was first divided into millions of finite volume mesh elements (surface element size ~ 100 µm; internal element size ~ 200 µm; three layers of boundary layer mesh [whenever possible] with an initial element's size of 50 µm and 10% growth rate). Regions with small features (e.g., edges of the delamination flaps, catheter, etc.) were subjected to further local mesh refinement based on local curvature. The total mesh elements for the 3D model with and without the catheter were 6,932,363 and 4,652,955, respectively.

An in-house solver with an extension for a non-Newtonian viscosity model based on the open-source CFD package (v7, The OpenFOAM Foundation Ltd., London, UK) was used to perform blood flow simulation in the two models^31^. The solver handles blood rheology using the Quemada equation^32^, assuming 45% haematocrit and a uniform blood density of 1060 kg/m^3^. To mimic individual ovine heartbeat, time-varying blood velocity was estimated by recording the angiographic frame-by-frame contrast filling along the model’s centreline (Fig. S3). The final time-varying velocity waveform was curve-fitted and discretised with a time-step size Δt = 0.001 s^12^. Each CFD simulation lasted for at least three cardiac cycles. ESS and OSI were calculated in the last cycle^33,34^, and were reported at critical time points (~ 1/15 s apart) based on the coronary angiogram cine rate.

### Statistical analysis

All statistical analyses were performed using R statistical software (R Foundation for Statistical Computing Platform v4.02, Vienna, Austria). Continuous variables with non-normally distributed variables were reported as median and interquartile range (IQR). Wilcoxon Rank Sum test was used to compare the impact of the catheter on local haemodynamic parameters. A p-value < 0.05 was considered significant.

## Supplementary Information

Supplementary Information.

Supplementary Video S1.

## Data Availability

Request for original data presented in this study can be directed to the corresponding author.
