# Supplementary material for: An optical coherence tomography and endothelial shear stress study of a novel bioresorbable bypass graft
Source: Sci Rep. 2023 Feb 20;13:2941. doi: 10.1038/s41598-023-29573-1 (PMC9941467; doi:10.1038/s41598-023-29573-1)
Supplement: Supplementary file 1 — Supplementary Information. [file 41598_2023_29573_MOESM1_ESM.docx]

# Supplementary Information

**
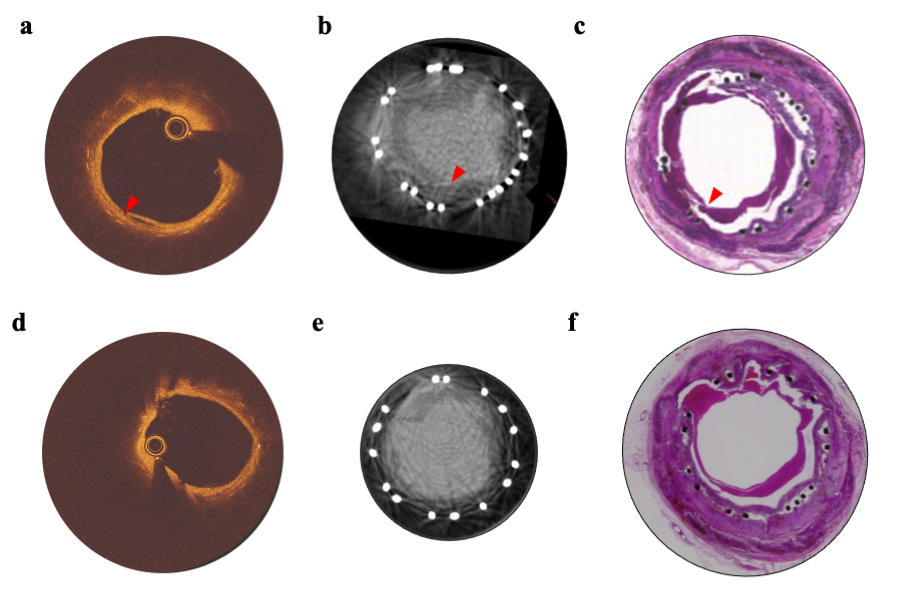
**

**Figure S1.** (**a**-**c**) display coregistered OCT image at 180 days, µCT and histology at 200 days proximal to the “flap” location. (**d**-**f**) are the OCT, µCT and histology distal to the “flap” location. OCT image (**a**) reveals a small piece of separated tissue (red arrow) proximally. And the corresponding µCT (**b**) and histology (**c**) also show a thin opening near this tissue location 20 days after (see red arrows). Histology at 200 days (**c** and **f**) also uncovers a dissected tissue ring around the conduit that was not present at the time of the OCT (**a** and **d**). It is unclear what led to this dissected ring in the histology. However, the conduit remained patent both proximally and distally as demonstrated by both µCT images and histology (**b**, **c**, **e** and **f**).


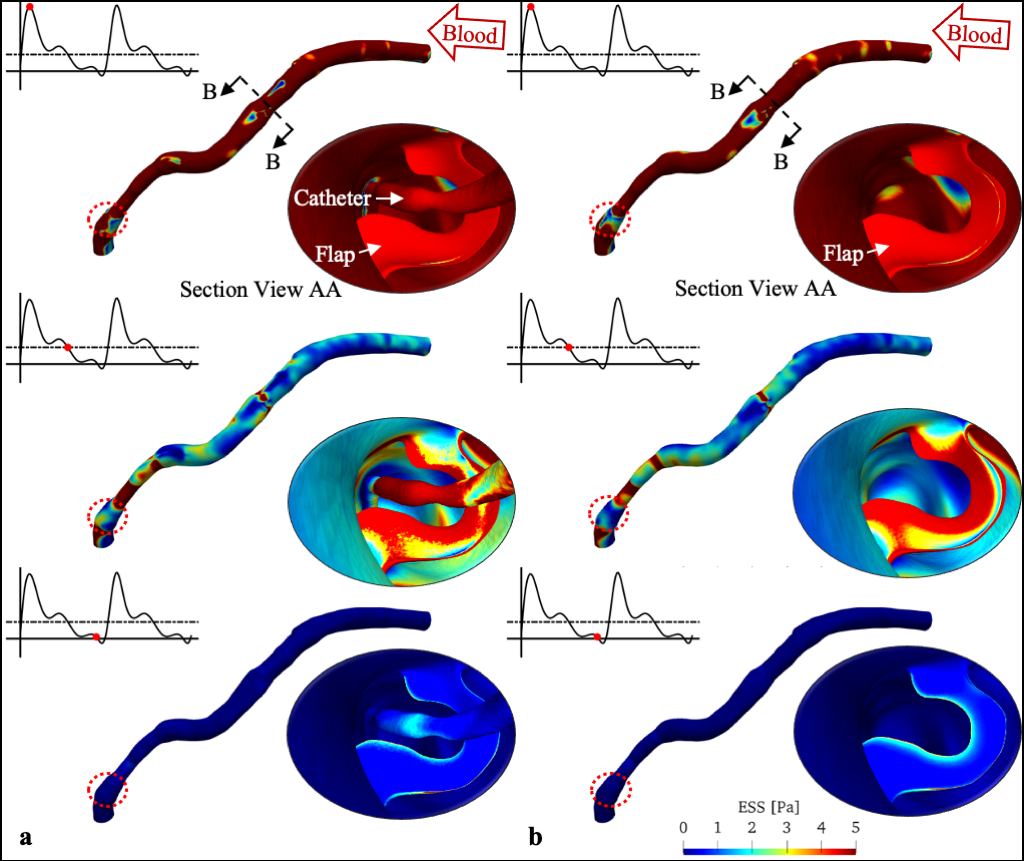


**Figure S2.** ESS patterns on the XABG at peak (time pt. 2), median (time pt. 6 [dashed line]) and minimum blood flow (time pt. 9). Insets show the section view B-B and the corresponding ESS pattern proximal to the “flap”. Red dotted circles highlight the focal region of low ESS at distal anastomosis. (**a**) CFD with the OCT catheter and (**b**) CFD without the OCT catheter. CFD = computational fluid dynamic simulation; ESS = endothelial shear stress; OCT = optical coherence tomography; XABG = Xeltis coronary artery bypass graft.


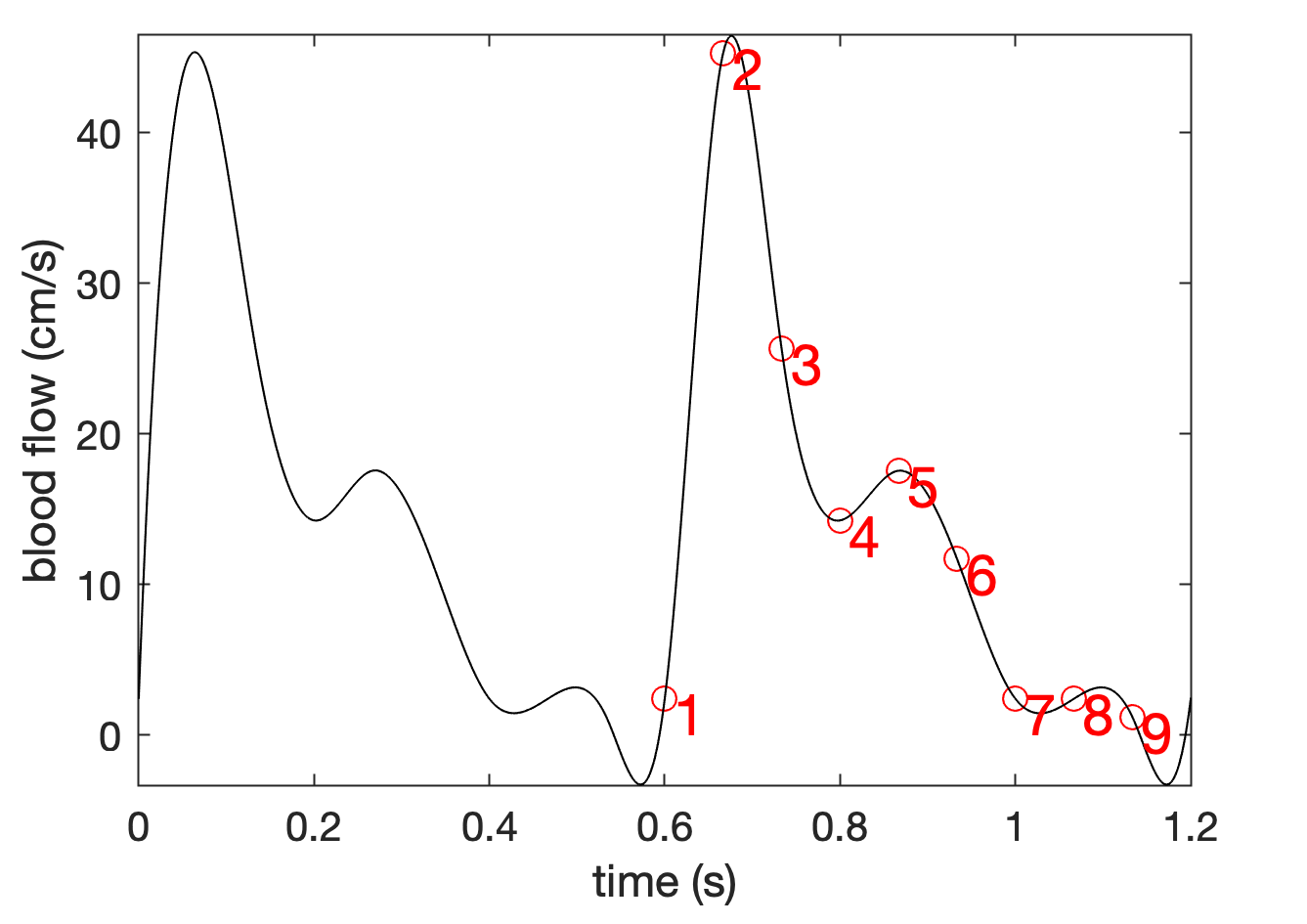


**Figure S3.** Reconstructed blood flow waveform by recording the angiographic frame-by-frame contrast filling along the model’s centreline. Red circles are the recorded blood flow velocity.

When considering the graft model, a significant difference in ESS was found for all time points (**Fig. S3**) except for time point 2 (End-QRS wave) and time point 4 (Mid T-wave) [**Supplementary Table S1**]. Further investigations by dividing the graft model into 4 separate ROIs: i) “flap”, ii) proximal 3 mm to the “flap”, iii) distal 3 mm to the “flap”, and iv) far-field (including both proximal and distal) segments revealed the impact of OCT catheter individually on each ROI. Besides the “flap” segment, all other ROIs showed significant differences in ESS with and without catheter presence (**Supplementary Table S2**). Both distal 3 mm and far-field ROIs constantly demonstrated lower ESS at all time points without the catheter presence. In contrast, proximal 3 mm ROI showed lower ESS in the systole when the flow is low (time pts 1, 8 and 9) but higher ESS during diastole (time pts 2 – 7) without the OCT catheter. In the “flap” segment, the difference in ESS was significant at all time points but not at time point 2 (peak flow) and time point 6 (median flow). Furthermore, withdrawing the OCT catheter increased ESS at all time points except point 1.

**Table S1. Comparisons of endothelial shear stress (ESS) of the full XABG with and without the OCT catheter over a cardiac cycle.**

| **Time Pt.** | **with catheter**  **ESS, Median (IQR)** | **without catheter**  **ESS, Median (IQR)** | **p-value** |
| --- | --- | --- | --- |
| 1 | 1.23 (0.45) | 1.17 (0.43) | <0.0001^****^ |
| 2 | 10.04 (12.81) | 9.98 (17.62) | 0.0916 |
| 3 | 5.17 (8.26) | 5.73 (11.30) | <0.0001^****^ |
| 4 | 2.60 (2.96) | 2.55 (3.81) | 0.1687 |
| 5 | 3.13 (3.44) | 2.88 (4.63) | <0.0001^****^ |
| 6 | 1.81 (2.45) | 1.70 (3.51) | <0.0001^****^ |
| 7 | 0.32 (0.40) | 0.35 (0.62) | <0.0001^****^ |
| 8 | 0.53 (0.31) | 0.50 (0.45) | 0.0023^**^ |
| 9 | 0.12 (0.21) | 0.10 (0.35) | <0.0001^****^ |

**Table S2. Comparisons of the endothelial shear stress (ESS) over a defined segment of the XABG with and without the OCT catheter over a cardiac cycle.**

| **Time Pt.** | **Flap ESS, Median (IQR)** | | | **Far field ESS, Median (IQR)** | | |
| --- | --- | --- | --- | --- | --- | --- |
|  | **with catheter** | **without catheter** | **p-value** | **with catheter** | **without catheter** | **p-value** |
| 1 | 1.06 (1.39) | 0.85 (1.15) | <0.0001^****^ | 1.24 (0.36) | 1.20 (0.31) | <0.0001^****^ |
| 2 | 30.25 (60.32) | 30.64 (59.42) | 0.2579 | 8.27 (6.48) | 7.14 (5.67) | <0.0001^****^ |
| 3 | 16.41 (28.38) | 17.19 (29.75) | <0.0001^****^ | 3.70 (5.06) | 3.17 (5.13) | <0.0001^****^ |
| 4 | 6.58 (10.51) | 6.76 (11.29) | 0.0221^*^ | 2.29 (2.06) | 1.96 (1.90) | <0.0001^****^ |
| 5 | 8.46 (14.64) | 8.47 (15.16) | <0.0001^****^ | 2.72 (1.95) | 2.30 (1.75) | <0.0001^****^ |
| 6 | 5.63 (9.20) | 5.84 (9.85) | 0.0727 | 1.53 (1.47) | 1.20 (1.35) | <0.0001^****^ |
| 7 | 0.89 (1.19) | 1.18 (1.55) | <0.0001^****^ | 0.27 (0.27) | 0.25 (0.26) | <0.0001^****^ |
| 8 | 0.78 (1.26) | 0.91 (1.19) | <0.0001^****^ | 0.51 (0.24) | 0.46 (0.17) | <0.0001^****^ |
| 9 | 0.48 (0.69) | 0.72 (0.97) | <0.0001^****^ | 0.10 (0.10) | 0.06 (0.07) | <0.0001^****^ |
|  |  |  |  |  |  |  |
| **Time Pt.** | **Distal 3mm ESS, Median (IQR)** | | | **Proximal 3mm ESS, Median (IQR)** | | |
|  | **with catheter** | **without catheter** | **p-value** | **with catheter** | **without catheter** | **p-value** |
| 1 | 1.16 (0.17) | 1.10 (0.20) | <0.0001^****^ | 1.48 (0.30) | 1.41 (0.45) | <0.0001^****^ |
| 2 | 10.96 (8.05) | 8.15 (8.76) | <0.0001^****^ | 13.50 (18.75) | 14.07 (33.89) | <0.0001^****^ |
| 3 | 6.52 (4.54) | 4.68 (4.41) | <0.0001^****^ | 6.07 (9.44) | 6.74 (15.70) | <0.0001^****^ |
| 4 | 2.81 (1.90) | 1.82 (1.55) | <0.0001^****^ | 3.07 (4.04) | 3.56 (7.58) | <0.0001^****^ |
| 5 | 3.25 (2.08) | 2.16 (1.75) | <0.0001^****^ | 3.91 (4.89) | 3.99 (8.77) | <0.0001^****^ |
| 6 | 2.21 (1.52) | 1.31 (1.05) | <0.0001^****^ | 2.21 (3.20) | 2.32 (5.76) | <0.0001^****^ |
| 7 | 0.35 (0.28) | 0.20 (0.15) | <0.0001^****^ | 0.21 (0.42) | 0.24 (0.85) | <0.0001^****^ |
| 8 | 0.52 (0.18) | 0.38 (0.16) | <0.0001^****^ | 0.62 (0.49) | 0.57 (0.48) | <0.0001^****^ |
| 9 | 0.13 (0.19) | 0.12 (0.08) | <0.0001^****^ | 0.12 (0.27) | 0.08 (0.28) | <0.0001^****^ |

**Table S3. Comparisons of the oscillatory shear index (OSI) of the full XABG with and without the OCT catheter.**

| **with catheter** OSI, Median (IQR) | **without catheter** OSI, Median (IQR) | p-value |
| --- | --- | --- |
| 0.0238 (0.0312) | 0.0263 (0.0379) | <0.0001^****^ |

**Table S4. Comparisons of the oscillatory shear index (OSI) over a defined segment of the XABG with and without the OCT catheter.**

| **Flap OSI, Median (IQR)** | | | **Far field OSI, Median (IQR)** | | |
| --- | --- | --- | --- | --- | --- |
| **with catheter** | **without catheter** | **p-value** | **with catheter** | **without catheter** | **p-value** |
| 0.0135 (0.0422) | 0.0128 (0.0547) | <0.0001^****^ | 0.0259 (0.0300) | 0.0298 (0.0328) | <0.0001^****^ |
|  |  |  |  |  |  |
| **Distal 3mm OSI, Median (IQR)** | | | **Proximal 3mm OSI, Median (IQR)** | | |
| **with catheter** | **without catheter** | **p-value** | **with catheter** | **without catheter** | **p-value** |
| 0.0175 (0.0305) | 0.0269 (0.0640) | <0.0001^****^ | 0.0198 (0.0112) | 0.0188 (0.0127) | <0.0001^****^ |
